# Machine learning-aided microRNA discovery for olive oil quality

**DOI:** 10.1371/journal.pone.0311569

**Published:** 2024-10-11

**Authors:** Mohammad Hossein Pakdel, Ali Akbar Asadi, Elahe Tavakol, Vahid Shariati, Mehdi Hosseini Mazinani

**Affiliations:** 1 Department of Plant Molecular Biotechnology, National Institute of Genetic Engineering and Biotechnology (NIGEB), Tehran, Iran; 2 Department of Plant Production and Genetics, Shiraz University, Shiraz, Iran; Nuclear Science and Technology Research Institute, ISLAMIC REPUBLIC OF IRAN

## Abstract

MicroRNAs (miRNAs) are key regulators of gene expression in plants, influencing various biological processes such as oil quality and seed development. Although, our knowledge about miRNAs in olive (*Olea europaea* L.) is progressing, with several miRNAs being identified in previous studies, but most of these reported miRNAs have been predicted without the aid of a reference genome, primarily due to limited genome accessibility at the time. However, significant knowledge gaps still need to be improved in this area. This study addresses the complexities of miRNA detection in olive, using a high quality reference genome and a combination of genomics and machine learning-based methods. By leveraging random forest and support vector machine algorithms, we successfully identified 56 novel miRNAs in olive, surpassing the limitations of conventional homology-based methods. Our subsequent analysis revealed that some of these miRNAs are implicated in the regulation of key genes involved in oil quality. Within the context of oil biosynthesis pathways, the novel miRNA Oeu124369 regulates fatty acid biosynthesis by targeting acetyl-CoA acyltransferase 1 and palmitoyl-protein thioesterase, thereby influencing the production of acetyl-CoA and palmitic acid, respectively. These findings underscore the power of machine learning in unraveling the complex miRNA regulatory network in olive and provide a high quality miRNA resource for future research aimed at improving olive oil production by exploring the target genes of the identified miRNAs to understand their role and their biological processes.

## Introduction

A substantial variety of endogenous non-coding RNAs (ncRNAs) are microRNAs (miRNAs) that regulate gene expression at the post-transcriptional level through the degradation of target mRNAs [1] and translation inhibition [2]. Plant miRNAs are regulators of different physiological processes, growth and development, meristem differentiation, hormone signaling, signal transduction, and response to biotic or abiotic stresses [3, 4]. Additionally, each plant species contains a large number of less conserved miRNAs, indicating the potential for miRNAs to have a functional impact on almost every aspect of plant life [4]. Therefore, understanding the function of the plant miRNAs appears to be crucial for further progress in breeding programs. Identification of miRNAs is challenging because miRNA families sometimes only differ in a single nucleotide. Therefore, it can be said that new methods such as machine learning are more accurate than previous methods and identify novel miRNAs at a lower cost and faster. For this reason, machine learning has been used to identify new miRNAs and their target genes in different plants families and species [5–9]. On the other hand, increasing the specificity of prediction algorithms by reducing the number of false-positive miRNA predictions is the most challenging part of machine learning models [10]. In a study performed by Douglass et al., (2016), Bayesian classifier was used to identify plant miRNAs in Arabidopsis, rice, soybean, and peach. In a separate study conducted by Williams et al. (2012), 18 plant species were utilized for miRNA prediction using a decision-tree model that was developed through supervised machine learning. In research carried out by Meng et al. (2014), a novel support vector machine-based classification model was developed to predict real and pseudo-plant pre-miRNAs along with their miRNAs. The model achieved an accuracy of around 90% when tested on plant datasets from nine different plant species.

Olive (*Olea europaea* L.) is particularly important for its balanced fatty acids and phenolic compounds. Due to the high economic value of olive oil, the availability of complete genomic information can be of great value, and therefore supplementing the available information regarding miRNAs can be a part of this goal. Research on miRNA in olive trees is currently insufficient, with only a few miRNAs identified in the available studies [11–13]. An initial study examined lateral buds of olive trees at two developmental stages and identified 18 known miRNA families [11]. In a study conducted on *Olea europaea*, researchers identified 135 miRNAs from 22 miRNA families in both mature and immature fruits, as well as leaves [12]. These miRNAs showed distinct expression patterns in various tissues and growth stages, emphasizing their regulatory function in controlling the transition between reproductive and vegetative phases [12]. In a different study, Guo et al. (2020) used a novel methodology to finally identify additional miRNAs by reanalyzing the data generated by Yanik et al. (2013) and Donire et al. (2011). In other species, the miRNAs are shown to control oil quality and seed development and investigated in soybean (*Glycine max*) and canola (*Brassica napus*) [14–16]. Wang et al. (2016) discovered that some miRNAs found in *Brassica napus* regulate functional genes such as 3-ketoacyl-ACP synthase and 3-ketoacyl-ACP reductase, which are directly involved in fatty acid biosynthesis. Moreover, Koerbes et al. (2012) predicted that miRNA target genes encode a diverse set of proteins involved in seed development and energy storage in *Brassica napus* [15]. In a study conducted by Song et al., (2011), 38 known miRNAs and 8 new miRNAs were discovered which may function in soybean seed development.

Despite advancements in miRNA research across plant species [17], our comprehension of miRNA identification and function in olive remains nascent. A review of the literatures shows that the miRNAs in olives have not been fully identified. On the other hand, a significant void exists in our knowledge of novel miRNAs and their regulatory roles in olive development and fruit quality. This study aims to bridge this gap by employing sophisticated computational approaches to identify and characterize novel miRNAs in olive. Given the presence of the olive reference genome [18] and transcriptome data from different tissues and developmental stages, we employed 12 miRNA libraries to detect known and potential new miRNAs with high confidence for the first time. Furthermore, to better understand the identified miRNA’s role in olive oil quality, we employed machine learning to identify miRNA’s target genes and also the regulatory roles of these miRNAs in shaping olive oil quality. The results of the present research can help to complete the olive annotation and clarify some of the unknown regions of the genome.

## Methods

### Data collection and preprocessing

Raw miRNA-seq data from three experiments (PRJNA184000, PRJNA137457 and PRJNA413783) were obtained from SRA database. Totally, these data contain 12 samples collected from leaves, fruits and lateral buds at different developmental stages (Table 1).

**Table 1:** The information of three experiments used in miRNAs prediction.

Project accession	Libraries	Platform	Tissue	Reference
**PRJNA184000**	6	Illumina	Fruit	[12]
			Leaves	
**PRJNA137457**	2	454 GS FLX	Lateral buds	[11]
**PRJNA413783**	4	Illumina	Fruit	[13]

The quality of raw miRNA-seq data was evaluated by using FastQC (0.11.9) [19] and trimmed by using Cutadapt (1.15) for removing adaptors and also low-quality bases from the end of reads. FastQC was utilized after each trimming to assess the properties of processed reads and to validate trimming efficiency. Based on the quality control results, the PRJNA137457 project data was lower than the other two data series that were sequenced using Illumina sequencing, and therefore needed more trimming.

### The miRNA detection

In the currents study, two methods were used for miRNAs detection: ShortStack and machine learning based approach. The ShortStack (3.x) [20] (https://github.com/MikeAxtell/ShortStack/releases) performs alignment, annotation, and quantification of expressed small RNAs. Therefore, miRNA-seq data in fastq format and the olive reference genome (OE9) [21] were given as input to ShortStack. The ShortStack employs bowtie to find all potential best-matched alignments for each read in the alignment process as the first step and then using default values and a user-adjustable limit of 50 alignments per read. After that, ShortStack will determine the likelihood of each alignment. The position of miRNAs on olive genome was determined by ShortStack. Regarding available machine learning algorithms Random Forest exhibit superior performance in miRNA detection due to their inherent robustness to overfitting and capacity to capture intricate feature interactions. Unlike SVMs, which often struggle with complex datasets, Random Forests can effectively handle the multifaceted nature of miRNA sequence and structural data, leading to enhanced prediction accuracy and reduced false positives. This capability is particularly advantageous when dealing with noisy or high-dimensional miRNA datasets. Given the established strengths of Random Forest algorithms in handling complex biological data, we opted to employ the state-of-the-art BrumiR package for our analysis [22]. BrumiR extracts key features from the miRNA sequences, including sequence length, GC content, minimum free energy of secondary structure, and the presence of known miRNA motifs. These features are used to train the random forest model and performance are evaluated using accuracy, precision, recall, and F1-score. A combined approach utilizing the statistical rigor of ShortStack and the predictive power of BrumiR provided us a comprehensive framework for miRNA identification. By capitalizing on the strengths of both methodologies, we will be able to expand the repertoire of identified miRNAs, enhancing the accuracy and depth of our analysis.

The position of miRNAs on olive genome was determined by ShortStack. We used the R software’s RIdeogram package (https://github.com/TickingClock1992/RIdeogram) (Hao et al., 2020) to show the distribution of detected miRNAs on olive chromosomes.

### Mapping and clustering miRNAs

To discover the expression level of each miRNA in each sample we mapped clean miRNA-seq reads against predicted miRNAs using Bowtie1 [23] and the number of each miRNAs were counted by Samtools [24]. Moreover, a BLAST search against known miRNAs downloaded from miRbase was conducted using blastn in NCBI-blast package.

### The miRNAs target detection and enrichment analysis

psRNATarget (http://plantgrn.noble.org/psRNATarget/) [25] was implemented with default parameters for detecting the candidate target of miRNAs. The OE9 [21] cDNA sequences of olive [26] was used to identify possible target of miRNAs. The resulted targets were graded based on their scores, from 0 to 5 with a range of changes of 0.5, and only grades equal to or less than 2.5 were accepted and used in the next steps (S1 Table). To complement the psRNATarget predictions, we employed the miTAR tool, a machine learning-based approach for miRNA target prediction. miTAR utilizes a support vector machine (SVM) to identify potential target sites based on sequence and structural features. We used miTAR with default parameters to predict miRNA targets in olive. By combining the results from both psRNATarget and miTAR, we aimed to increase the confidence in target identification. (S2 Table) [27]. Leveraging both miTAR and psRNATarget for miRNA target prediction offers a synergistic approach. While psRNATarget excels in thermodynamically stable target site prediction, miTAR’s machine learning framework expands the search space for potential regulatory interactions. This complementary strategy not only enhances the confidence in predicted targets through concordance analysis but also has the potential to unveil novel miRNA-target relationships, thereby providing a more comprehensive understanding of miRNA-mediated gene regulation. The pathway enrichment analysis was conducted using in house pipeline scripts and related pathways were selected for more investigation.

### Expression profile of miRNA targets

The genes expression pattern in fruit and leaf tissues were determined based on RNA-seq data (Ahmed et al. 2012) (PRJNA556567) as a gene expression atlas to identify the expression profile of miRNA targets (S3 Table). The quality control of raw Illumina RNA-seq reads was evaluated by FastQC software (0.11.8) (http://www.bioinformatics.babraham.ac.uk/projects/fastqc/). The raw reads were trimmed by Trimmomatic software (0.32) by discarding adaptors, ambiguous nucleotides, low-quality (<20), and short-length reads (<50 nt) for all the experiments. FastQC was utilized after each trimming to assess the properties of processed reads and to validate trimming efficiency. The clean reads were mapped onto the olive genome as the reference (OE9) using the Hisat2 [28]. The aligned reads were sorted by position using the Samtools. The read counts were calculated using the HTSeq [29] to estimate the count of uniquely mapped reads for each of the experiments. Finally, the differential expression analysis was carried out by DESeq2 [30] with default hypothesis testing method (Wald test) and false discovery rate (FDR) <0.05 was used to find the differentially expressed genes in tissues and development stages comparisons.

## Results and discussion

Twelve samples collected from different tissues and developmental stages, were used to predict miRNAs in olive. These miRNA-seq samples contain 265,098,547 reads with an average of 22,091,545 reads per sample (S4 Table). In the initial method, we detected 150 miRNAs in olive using ShortStack (S5 Table) and additionally 5476 miRNAs were identified through a machine learning approach (S6 Table). The integration of machine learning into miRNA identification has emerged as a pivotal advancement, surmounting the inherent limitations of traditional homology-based approaches. Unlike sequence alignment methods, which heavily rely on the availability of homologous sequences, machine learning algorithms excel in discerning intricate patterns within miRNA sequences, enabling the discovery of novel miRNAs with minimal sequence similarity [31]. Moreover, traditional methods often struggle with computational efficiency when handling vast datasets, a challenge effectively addressed by the scalability of machine learning models. Recognizing these limitations, we employed a dual-pronged machine learning strategy for miRNA prediction. The BrumiR pipeline, incorporating both random forest and support vector machine (SVM) algorithms, was instrumental in capturing the complexity of miRNA sequence features. Random forest, renowned for its ensemble learning approach, proved adept at handling the high-dimensional and noisy nature of miRNA sequence data, while SVM’s kernel-based techniques offered flexibility in modeling complex relationships between features [32, 33]. By combining these complementary algorithms, we aimed to maximize our ability to identify both known and novel miRNAs, thereby expanding the breadth of our miRNA repertoire. This machine learning-driven approach offers several advantages over traditional methods. Firstly, it enables the identification of miRNAs with low sequence similarity to known miRNAs, expanding the discoverable miRNA space. Secondly, the ability to handle large datasets efficiently allows for the analysis of complex miRNA expression patterns. Thirdly, the incorporation of multiple machine learning algorithms enhances the robustness and accuracy of miRNA prediction. The judicious selection of machine learning algorithms was paramount to the success of this study. Random forest and support vector machines were chosen due to their complementary strengths in handling complex biological data. Random forest, with its ensemble nature, is robust to overfitting and effectively captures intricate patterns within miRNA sequences. Conversely, support vector machines excel in identifying optimal decision boundaries, particularly in high-dimensional spaces, such as those encountered in miRNA analysis [34, 35]. We compared our predictions with established miRNA databases (e.g., miRBase) which these validation steps were crucial in establishing the credibility of our findings. The BLAST of detected miRNAs against the miRbase database (hairpin and mature) showed 84 significant hits. However, 66 miRNAs did not have any hits in miRbase database. Consequently, we classified all the detected miRNAs into 3 groups. The first group of miRNAs shown by one-star (*), are the miRNAs that have hits in the mature dataset of miRbase database. The second group of miRNAs shown by two-stars (**), contain miRNAs that have not hit in mature database but have hits in the hairpin precursor sequences of miRbase database. If the miRNAs did not have any hit in any of mature and hairpin databases in miRbase, it was set as third group with three-stars (***), and were considered as putative novel miRNAs. The two experiments (PRJNA184000 and PRJNA137457) in the present study were previously used by Guo et al [36], and the distinction between the current investigation and their research lies in the approach for detecting miRNAs, with their findings indicating the identification of 266 miRNAs through the utilization of the mirdeep2 software package. The results of the present study demonstrated that 70 miRNAs from 150 identified miRNAs were also reported by Guo et al., (2020) in *Olea europaea*. Comparing the results of the current study with the studies of Donire et al., (2011) and Yanik et al., (2013) shows that 56 novel miRNAs in the present investigation were not reported in previous studies. Therefore, according to the obtained results, it can be said that totally 56 new miRNAs have been identified in olive. Their information and potential target genes are listed in Table 2 and S5 Table. The results demonstrated that tissue, developmental stage of the samples and miRNAs identification approach have impact on the number of miRNAs [37–39].

**Table 2:** The information of 56 putative novel miRNAs in olive.

miRNA	miRNA sequence	Chromosome position	Targets number
**Oeu7865**	TGGGAAAACAGGGGGGCGGTCA	2:10464576–10464706	4
**Oeu207142**	AAGTGACCCTGACAGACTAGCATA	1:8059–8268	7
**Oeu5499**	GCCATGTAGGACGATTACCGATAG	1:31586249–31586447	3
**Oeu8938**	ATGGGTGGTAGTTACGAACGTGAG	2:18108667–18108946	1
**Oeu9273**	ACGACTGCTGTAGTGAGCGACTGC	2:20008492–20008699	1
**Oeu38941**	TGTCATGATTTGAATAGTTCGGAG	7:10944026–10944175	6
**Oeu53669**	AGTGACTCCGTGGAAGCATTTTAC	11:4257463–4257730	1
**Oeu62331**	GAGGTGTAAAGTGGGTTGGGTTGG	12:21576103–21576167	7
**Oeu66021**	AAGCAAGACCGAACATGACCA	13:15339075–15339297	6
**Oeu71543**	TCCATTTTGGAGAGTTTGAGCATT	15:2927120–2927288	4
**Oeu91814**	GGAGGGGCGGCTGTGTCAATTCA	21:4348323–4348597	3
**Oeu95050**	AGGAGAGTGTACACCGGAATGTCA	22:7700992–7701183	1
**Oeu98360**	ACTTTTTGGTTGTTTGATTGCAAG	1:533490–533775	4
**Oeu99834**	ATAAATTAAGTATCGGTGTTTAGG	1:931241–931494	1
**Oeu115815**	ATACAGCAACATGGGGACTGATA	1:438834–439079	1
**Oeu124369**	GATGGATGAAATTGGTATGGGTGA	1:544236–544476	21
**Oeu142324**	AGCCAATATCGACGAATGAGCAAG	1:194900–195052	1
**Oeu145482**	AAAATCCTTCTGGCAGCTCGGCAT	1:198268–198480	1
**Oeu184651**	ACTAGTGCAGGTGGACAACAT	1:60158–60260	3
**Oeu192090**	AATGAATTATTGGTGTACAGCAAG	1:14613–14900	2
**Oeu197013**	AAATGATCTAATGGACATCGGTGT	1:20622–20867	2
**Oeu197612**	AGGAGAGTGTATACCGAACTGTAA	1:42920–43171	1
**Oeu365**	GGTTGAGAGTTGTAGGAAATG	1:3147594–3147745	5
**Oeu6327**	GCTCACCCTCTTTCTGTCACC	2:32098037–32098159	1
**Oeu9135**	TCGGTTGGTGCAGTTCGGGAG	3:23416308–23416413	3
**Oeu9740**	CGGTGCCACGCTGTGTGCGAC	3:30418789–30419002	1
**Oeu11313**	ACACCCTTCGGCACACCAAATTAT	4:13119963–13120122	5
**Oeu12130**	ATCGATCTATGTGGCATTGAGGTC	4:21716090–21716200	9
**Oeu21457**	TTAGATTCACGCACAAACTCG	7:4580674–4580834	6
**Oeu27159**	GTATTGGAAGACTTGTGGACC	10:9469652–9469785	7
**Oeu28501**	GGTGCAATGGGGTGACGCCGAGA	11:2269904–2270190	3
**Oeu33233**	AGCTCAACCAACTTTACACCTCTA	12:21576092–21576173	5
**Oeu34586**	GTTCGCTTCCACCACTTGAAG	13:9838519–9838702	7
**Oeu39167**	CGTTTTGGATCGGCCTTGCGCT	15:13278082–13278363	1
**Oeu49787**	AGATGCTGGTGTTGGTGATCGCG	21:10246955–10247173	7
**Oeu52228**	GCACGTCGGACATTCTGCTAGAGA	CA.1:1090272–1090390	3
**Oeu52650**	TGCTCCATATCCAGTCCTGAG	CA.1:507480–507584	5
**Oeu53653**	AGCCCAACCAACTTTACACCTCTA	CA.1:1103886–1104092	7
**Oeu68016**	ACAGAATACTCACATGCAGGGCTC	CA.1:438262–438471	4
**Oeu68174**	TGAGGGGGTTGTATGACATGATG	CA.1:32063–32174	3
**Oeu69526**	GGTGCAATGGGGTGACGCCGAGA	CA.1:251041–251205	4
**Oeu70966**	ATGAATCAAGGGTCCACTATCACC	CA.1:115920–116110	1
**Oeu71318**	ACCGCAGTTGCCTTTCGTGATATA	CA.1:237319–237618	2
**Oeu72434**	AGTGCCATCTCTTCTGTGACT	CA.1:411613–411699	-
**Oeu72818**	CACGTGCCTGTCTTCCCCATC	CA.1:399793–399907	2
**Oeu76531**	TTACAAAATTAAGAAGTGGCGGCC	CA.1:347863–347965	9
**Oeu77844**	CATGGTGGGCATTATAACTCA	CA.1:195016–195097	10
**Oeu82910**	TTTGATGTCAGCATTCCCTCC	CA.1:40851–41070	5
**Oeu83091**	CCTTTTCTTTCTGTACTTTGGG	CA.1:241019–241317	34
**Oeu86548**	TGATATGCCATGAACAATGATC	CA.1:2460–2535	6
**Oeu89051**	GACAAACTCGACACTTGGCGGCCC	CA.1:114852–115073	1
**Oeu93033**	GTACACCGGAGTGTTAACCTC	CA.1:116082–116264	1
**Oeu94849**	ATGGGATGTCACGATGAATGA	CA.1:179631–179812	4
**Oeu100566**	GTTCAAGAAAGCTGTGGGACA	CA.1:31367–31476	12
**Oeu100857**	CTGGGCGACCTGATGAGGTGGC	CA.1:84973–85150	-
**Oeu109699**	TCTCGTACTACATGGAATGCT	CA.1:9521–9804	2

**Fig 1:**
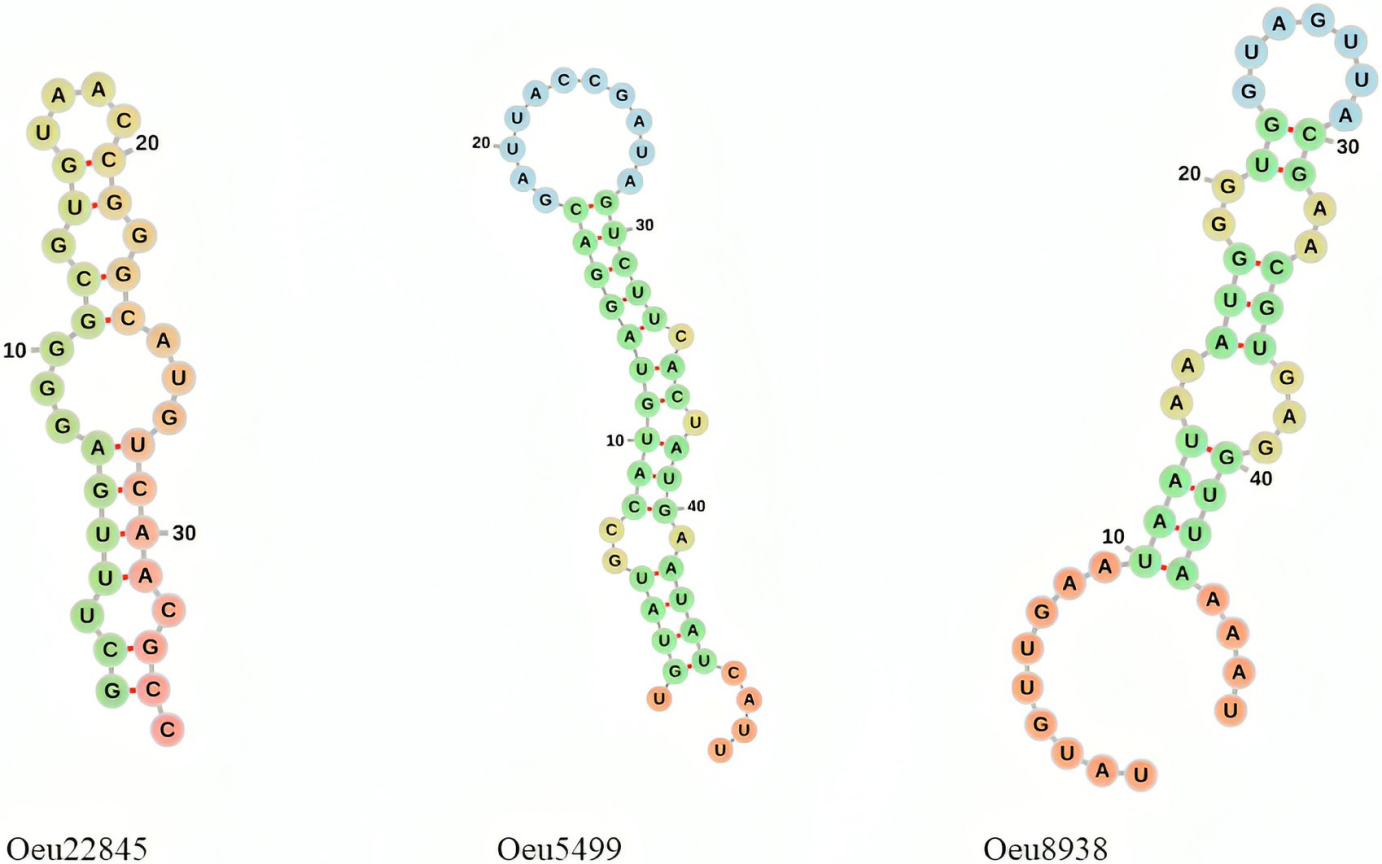
The secondary structure of tree novel olive miRNA. This secondary structure shows the hairpin structure of predicted miRNAs.

To further validate the identified microRNAs, we investigated their secondary structures. The secondary structure of microRNAs (miRNAs) is a critical determinant of their function and stability [40]. Understanding miRNA secondary structure is crucial for accurate detection, functional characterization, and the development of therapeutic strategies targeting miRNAs [41]. We have modeled the secondary structures of the three putative miRNAs we identified, and these are shown in Fig 1.

**Fig 2:**
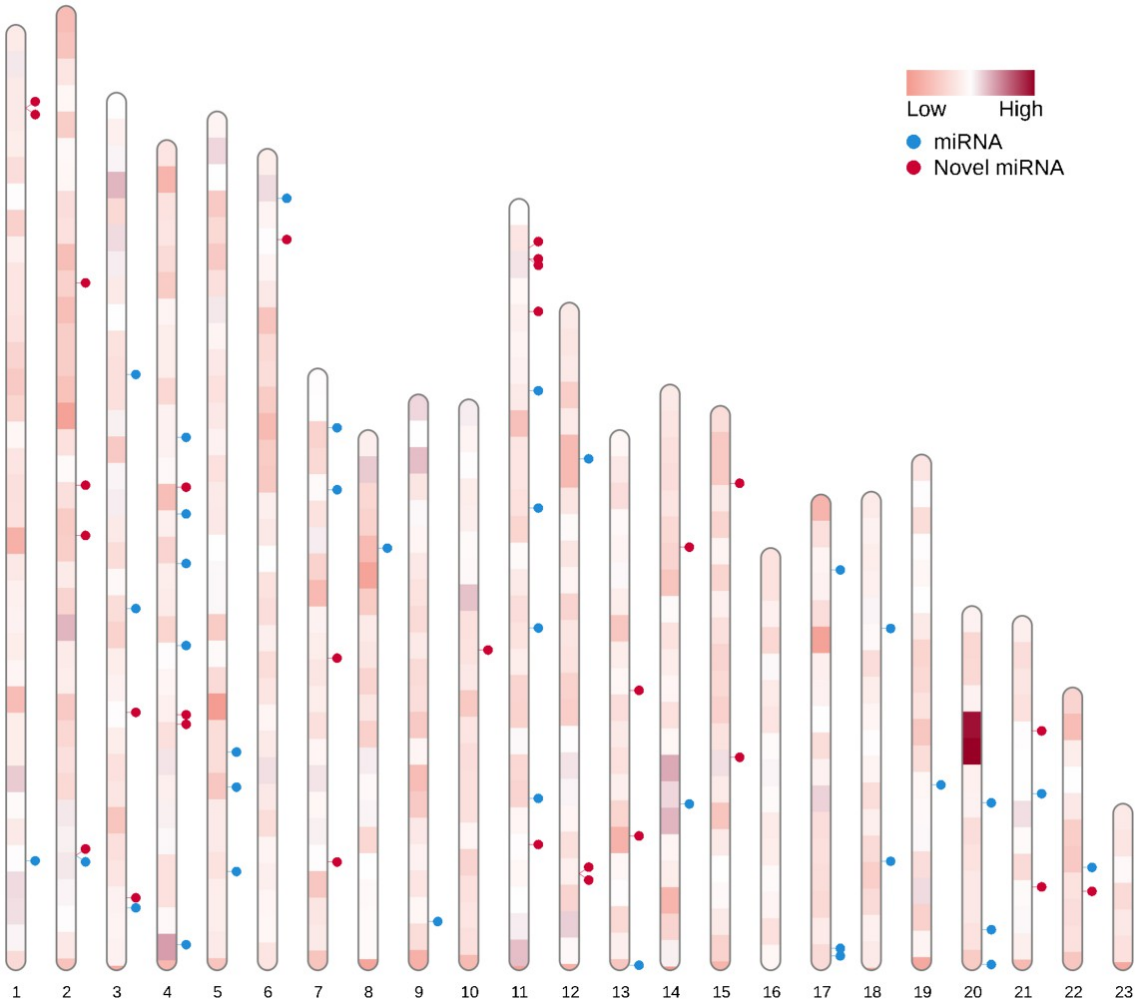
The distribution of detected miRNAs on olive chromosomes. 43% of identified miRNAs were localized on chromosomes and chromosomes of 16 and 23 had no miRNAs, while the others had similar numbers.

The chromosomal distribution of all detected miRNAs was graphically depicted using the RIdeogram package (Fig 2). Among the 150 detected miRNAs, 65 miRNAs (43%) were mapped on chromosomes and the rest of them were mapped on contigs. The detailed information of miRNAs distribution on different chromosomes is shown in S7 Table. According the results chromosomes 16 and 23 were microRNA-free and other miRNAs are almost equally distributed on other chromosomes.

### Predicted miRNA targets and pathway detection

**Fig 3:**
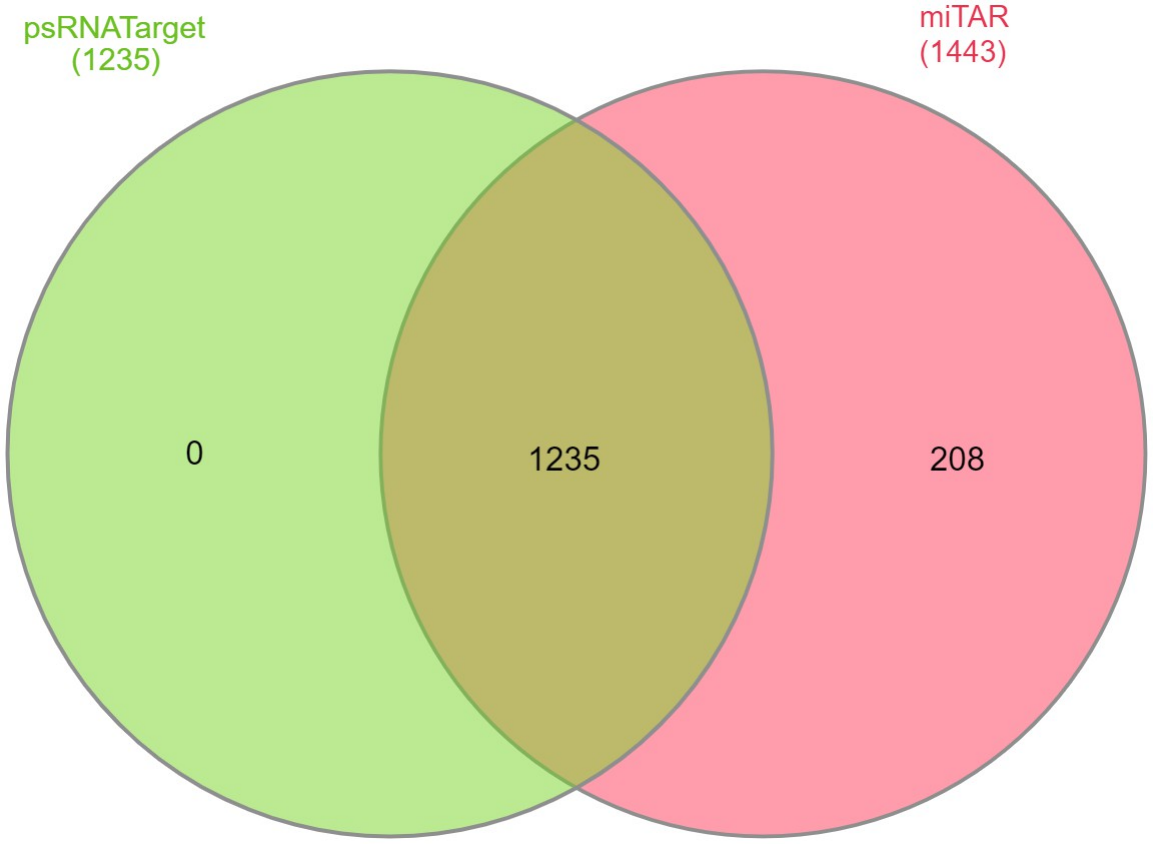
The gene IDs targeted by miRNAs that are shared between psRNATarget and miTAR. A significant overlap of 1235 genes was observed in the target predictions of psRNATarget and miTAR.

The miRNA-regulated genes control a variety of biological and metabolic processes such as leaf, stem, root, and flower development and responses to abiotic and biotic stresses [42, 43]. Moreover, one miRNA can target more than one regulatory gene [44]. Therefore, characterization of a miRNA target is essential to provide a biological insight into each miRNA-mediated pathway. The prediction of targets for the all miRNAs was done by psRNATarget and 1235 targets were identified. In contrast, the machine learning method predicted 1443 targets, with 1235 of them being in common with psRNATarget (Fig 3).

The pathway enrichment analysis was performed for the targets and 72 metabolic pathways were identified (S8 Table). The evaluation of 10 top metabolic pathways shows a significant number of putative novel miRNAs are involved in these pathways. The fatty acid elongation and fatty acid metabolism are critical pathways on the fatty acid biosynthesis and also flavonoid biosynthesis, flavonoid biosynthesis and phenylpropanoid biosynthesis are important pathways related to rare compounds in olive oil quality [45]. Hormone signal transduction is also a crucial process in all plant species.

### Fatty acid biosynthesis related pathways

Providing carbon to create the carbon skeleton of fatty acids is one of the stages of fatty acid biosynthesis, which is provided from various sources. The pathway of galactose metabolism is one of the main pathways that can help to supply the carbon skeleton of fatty acids. In the galactose pathway, UDP-Galactose must be produced for the synthesis of galactinol as a precursor in oligosaccharides production. The UDP-Galactose is generated from two distinct metabolites and enzymes [46]. One of the routes identified is known as Leloir pathway [47], catalyzed by UDP-glucose 4-epimerase enzyme [48]. Previous studies indicate that higher plants either lack or possess limited activity of the UDP-glucose 4-epimerase enzyme associated with the Leloir pathway [48, 49]. The results of the present study show that miRNA Oeu21457 targets the UDP-glucose 4-epimerase (OE9A021881) enzyme and causes its inactivation, which can be one of the reasons for the lower activity of this pathway in UDP-Galactose production. The analysis of UDP-glucose 4-epimerase expression in the fruit and leaf tissues suggests that the Oeu21457 activity is likely higher in the fruit.

Acetyl-CoA as important compound in fatty acid metabolism, is supplied in plant cells in two ways: from pyruvate in the glycolysis pathway and in the second way with beta-oxidation of fatty acids. After the production of long-chain fatty acids, and process of beta-oxidation of fatty acids, acetyl-CoA is finally released [50]. The results of the meta-analysis conducted by Asadi et al, show that five enzymes are effective in the process of beta-oxidation of fatty acids, which include acyl-CoA oxidase, Enoyl-CoA hydratase, beta-hydroxyacyl dehydrogenase, long-chain-3-hydroxyacyl-CoA dehydrogenase, and acetyl-CoA acyltransferase [45]. Present study show the novel miRNA Oeu124369 targets the acetyl-CoA acyltransferase 1 enzyme (EC: 2.3.1.16) (ACAA1) (OE9A119317) in the path of beta-oxidation of fatty acids (Fig 2). The RNA-seq data analysis indicates that Oeu124369 expression is higher in the leaf compared to the fruit, resulting in lower ACAA1 expression in the leaf. This suggests that the biosynthesis of fatty acids in the olive leaf may be lower than in the fruit. Therefore, if one key enzyme in this pathway is deactivated, it can disrupt the synthesis of acetyl-CoA and subsequently impact the production of other fatty acids. Pye et al, also reported bna-miR395d, bna-miR395e, and bna-miR395f, which regulate ACAA1 are involved in fatty acid elongation. Furthermore, miR858 was found to target two other genes both encoding [51]. In another study conducted on *Brassica napus*, eight miRNAs were discovered which target genes that are involved in acetyl-CoA generation [52].

**Fig 4:**
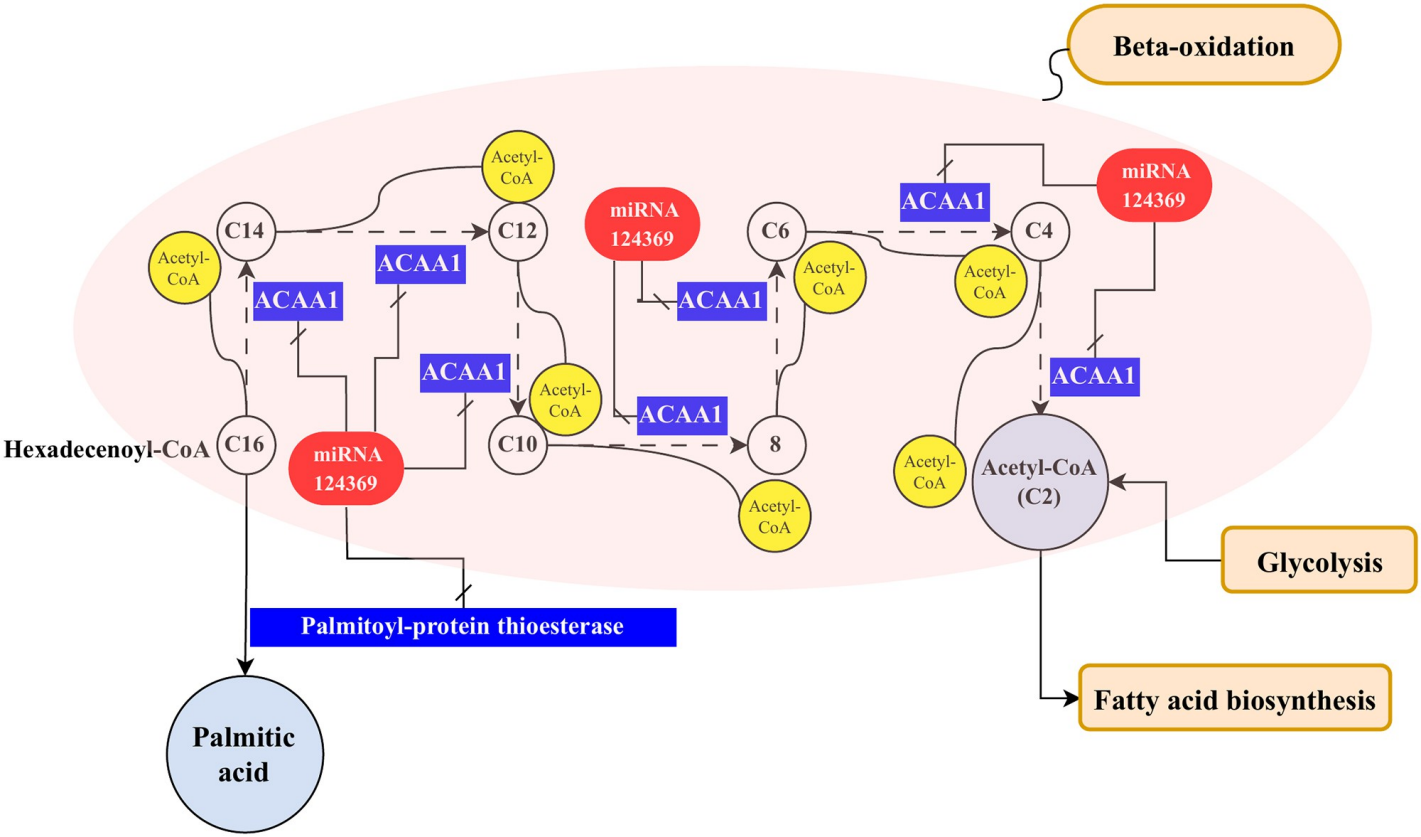
The inactivation of the acetyl-CoA acyltransferase 1 enzyme by the novel putative miRNA Oeu124369 leads to a decrease in Acetyl-CoA production and additionally, the deactivation of palmitoyl-protein thioesterase by the same miRNA results in a reduction in palmitic acid biosynthesis in the fatty acid elongation pathway.

Another target of miRNA Oeu124369 in the fatty acid metabolism pathway is palmitoyl-protein thioesterase (EC: 3.1.2.22) (OE9A102418) at the end of fatty acid elongation. In the fatty acid biosynthesis, palmitic acid is the first fatty acid produced during fatty acid synthesis [53]. The miRNA Oeu124369 targets the palmitoyl-protein thioesterase and reduced production of palmitic acid. The palmitoyl-protein thioesterase remove CoA from palmitoyl-CoA and produce the palmitic acid (Fig 4). Regarding the level of palmitic acid is one of the indicators of oil quality improvement, it can be said that the inactivity of palmitoyl-protein thioesterase enzyme by miRNA Oeu124369 is probably effective in regulating palmitic acid production and oil quality. In brinjal (*Solanum melongena*), Sme-miR529 binds to 14 different mRNAs, one of which is involved in palmitoyl hydrolase processing [54].

### Minor compounds related pathways

Phenolic compounds are significant components found in olive fruits. Research on the phenolic profiles in various olive tissues has revealed the presence of unique compounds. These compounds play a crucial role in determining the quality of olive oil by impacting its taste, contributing to bitter and pungent sensory characteristics, acting as primary antioxidants, and influencing the oil’s oxidative stability [55, 56]. In the biosynthesis of phenylpropanoids, key compounds like flavonoids, lignin, and verbascoside are synthesized. Intermediate compounds such as p-Coumaric acid and p-Coumaroyl-CoA play a crucial role in the production of these compounds in this pathway. A portion of the verbascoside structure is formed through the phenylpropanoid pathway when p-Coumaric acid is transformed into caffeic acid. Additionally, in an alternate pathway, p-Coumaric acid can be converted to p-Coumaroyl-CoA to generate flavonoids and lignin [57, 58]. The present study results show that miRNAs Oeu53211 and Oeu91814 target peroxidase (POD) (EC: 1.11.1.7) (OE9A091733), caffeic acid 3-O-methyltransferase (COMT) (EC: 2.1.1.68) (OE9A010735), and Shikimate O-hydroxycinnamoyltransferase (HCT) (OE9A103182) (EC: 2.3.1.133) enzymes in the pathway of phenylpropanoid biosynthesis, which can reduce lignin production. The RNA-seq analysis further validated that Oeu53211 expression is elevated in the fruit, suggesting that lignin production in this tissue may be lower, resulting in softer fruit tissue. In Chinese olive, there is a positive correlation between peroxidase enzyme expression and lignin content, which indicates that it is involved in lignin biosynthesis [59]. Lignin biosynthesis is catalyzed via a series of enzymes, including phenylalanine ammonia-lyase (PAL), 4-coumarate-CoA ligase (4CL), cinnamyl alcohol dehydrogenase (CAD), cinnamoyl-CoA reductase (CCR), and PODs. Therefore, peroxidase enzyme plays an important role in lignin polymerization and the Oeu53211 and Oeu91814 target the POD and have probably negative effects on lignin polymerization. On the other hand, in a study conducted by Alagna et al., (2012) peroxidase enzyme was introduced as an important enzyme in the phenolic compound degradation in olive fruits, which is especially expressed in the early stages of growth (45 days after flowering). Therefore, the identification of the Oeu53211 and Oeu91814 miRNAs that target the peroxidase enzyme can rule the level of polyphenols and their decomposition. It is noteworthy that increase of lignin level in the fruit tissue can have negative effects on the quality and tissue of fruit.

Squalene as another crucial compound in olive oil, has a positive impact on human health and also contributes to the oil stability by protection against oxidation. Squalene is produced in following of the terpenoid backbone biosynthesis pathway and by activity of the enzyme squalene synthase (EC: 2.5.1.21), farnesyl diphosphate is converted to squalene [60]. In sesquiterpenoid and triterpenoid biosynthesis, miRNA Oeu80058 targeted squalene synthase (OE9A101945), inactive the enzyme, and probably reduced squalene production. In high phenolic cultivars, the squalene synthase enzyme is expressed highly at 90 to 112 days after flowering while in low phenolic cultivars the squalene synthase has stable expression from 45 to 165 days after flowering and also is lowest than high phenolic cultivars [56, 61]. Therefore, this miRNA can reduce the quality of oil in some genotypes by inactivating an important enzyme in the production of squalene.

## Conclusion

Plant miRNAs play a crucial role as regulators of various physiological processes and understanding their functions is essential for advancements in breeding programs. Recently, machine learning methods have proven to be more accurate and cost-effective in predicting and identifying novel miRNAs compared to traditional methods. In the current study, machine learning was utilized for the first time to identify miRNAs and their target genes in olive. In the current investigation, 150 miRNAs were identified using ShortStack and 5476 miRNAs were identified through a machine learning approach. According to the obtained results, 56 novel miRNAs in the present investigation were not reported in previous studies. A total of 1235 targets were identified and 72 metabolic pathways were recognized. The carbon skeleton essential for fatty acid biosynthesis is derived from various sources, including the galactose metabolism, which leads to the formation of UDP-Galactose crucial for fatty acid synthesis. The miRNA Oeu21457 targets UDP-glucose 4-epimerase, potentially influencing the production of UDP-Galactose. Acetyl-CoA, a pivotal component in fatty acid metabolism, is produced through glycolysis from pyruvate and beta-oxidation of fatty acids. The novel miRNA Oeu124369 targets acetyl-CoA acyltransferase 1, impacting fatty acid production. Additionally, Oeu124369 targets palmitoyl-protein thioesterase, affecting palmitic acid production. These findings provide insights into miRNA regulation in fatty acid biosynthesis, potentially enhancing oil quality. Phenolic compounds are crucial components in olive fruits, directly impacting olive oil quality by contributing to its flavor and serving as primary antioxidants for stability. These compounds, responsible for bitter and pungent notes, are essential for the overall flavor profile. Studies demonstrate the intricate biosynthesis of phenylpropanoids in olives, necessary for producing flavonoids, lignin, and verbascoside. The miRNAs Oeu53211 and Oeu91814 were observed to target enzymes in the phenylpropanoid pathway, potentially altering lignin production and fruit quality. Notably, lignin biosynthesis involves enzymes like peroxidases, crucial for polymerization. Squalene, another significant component in olive oil, influences both human health and oil stability. The miRNA Oeu80058 was found to target squalene synthase, impacting squalene production. Variations in squalene synthase expression at different growth stages are linked to phenolic content, indicating genotype-specific regulation of squalene production and oil quality. Understanding these processes can aid in enhancing olive oil quality and maintaining desirable fruit characteristics. Moreover, the identified miRNAs aid in improving the annotation process and elucidating certain obscure areas of the genome.

## Supporting information

S1 Table Identified possible targets of miRNAs. (XLSX)

S2 Table miRNA target prediction based on machine learning approach. (XLSX)

S3 Table Gene expression atlas of olive genes. (XLSX)

S4 Table Raw sample data. (XLSX)

S5 Table Predicted miRNA data using ShortStack. (XLSX)

S6 Table Predicted miRNA data using machine learning approach. (XLSX)

S7 Table The information of miRNAs distribution on olive chromosomes. (XLSX)

S8 Table Pathway enrichment analysis results. (XLSX)
